# Lumbar degenerative spinal deformity: Surgical options of PLIF, TLIF and MI-TLIF

**DOI:** 10.4103/0019-5413.62066

**Published:** 2010

**Authors:** Hwee Weng Dennis Hey, Hwan Tak Hee

**Affiliations:** Department of Orthopedic Surgery, 5 Lower Kent Ridge Road, Singapore 119074

**Keywords:** Degenerative spine, lumbar spine fusion, minimally invasive transforaminal fusion

## Abstract

Degenerative disease of the lumbar spine is common in ageing populations. It causes disturbing back pain, radicular symptoms and lowers the quality of life. We will focus our discussion on the surgical options of posterior lumbar interbody fusion (PLIF) and transforaminal lumbar interbody fusion (TLIF) and minimally invasive transforaminal lumbar interbody fusion (MI-TLIF) for lumbar degenerative spinal deformities, which include symptomatic spondylolisthesis and degenerative scoliosis. Through a description of each procedure, we hope to illustrate the potential benefits of TLIF over PLIF. In a retrospective study of 53 ALIF/PLIF patients and 111 TLIF patients we found reduced risk of vessel and nerve injury in TLIF patients due to less exposure of these structures, shortened operative time and reduced intra-operative bleeding. These advantages could be translated to shortened hospital stay, faster recovery period and earlier return to work. The disadvantages of TLIF such as incomplete intervertebral disc and vertebral end-plate removal and potential occult injury to exiting nerve root when under experienced hands are rare. Hence TLIF remains the mainstay of treatment in degenerative deformities of the lumbar spine. However, TLIF being a unilateral transforaminal approach, is unable to decompress the opposite nerve root. This may require contralateral laminotomy, which is a fairly simple procedure. The use of minimally invasive transforaminal lumbar interbody fusion (MI-TLIF) to treat degenerative lumbar spinal deformity is still in its early stages. Although the initial results appear promising, it remains a difficult operative procedure to master with a steep learning curve. In a recent study comparing 29 MI-TLIF patients and 29 open TLIF, MI-TLIF was associated with longer operative time, less blood loss, shorter hospital stay, with no difference in SF-36 scores at six months and two years. Whether it can replace traditional TLIF as the surgery of choice for degenerative lumbar deformity remains unknown and more studies are required to validate the safety and efficiency.

## INTRODUCTION

With an ageing population, surgeons face a new challenge of managing patients with age related medical problems. Degenerative spinal disease has become a greater problem than before. Its spectrum includes degenerative disc diseases, facet joint arthritis, spinal stenosis, degenerative spinal scoliosis and spondylolisthesis.^1^ Often, they present with back pain, radicular pain and stiffness resulting in numerous people unable to perform in their work and losing quality of life.^1^

We will focus our discussion on the surgical options of posterior *lumbar* interbody fusion (PLIF) and transforaminal *lumbar* interbody fusion (TLIF) and minimally invasive transforminal lumbar interbody fusion (MI-TLIF) for lumbar degenerative spinal deformities, which include symptomatic spondylolisthesis and scoliosis.

Conservative management has always been advocated for these patients prior to surgical management.^2^ Although properly selected patients are likely to benefit from surgery, under the circumstances of a non emergency situation, most patients prefer conservative approaches comprising of physiotherapy, acupuncture and lifestyle modifications. Surger y is indicated in patients with symptomatic spondylolisthesis and degenerative lumbar scoliosis.^3^,^4^

To meet the increasing demands and expectations of patients, more innovative surgical techniques have been developed. New techniques attempt to shorten operative time and achieve faster recovery with lesser operative complications. Therefore the earlier works of anterior/ posterior lumbar interbody fusion (ALIF/PLIF) have been challenged by the gradually evolved transforaminal approach. Further, increasing numbers of surgeons are continuously striving to improve operative results via minimally invasive techniques (MI-TLIF). We attempt to discuss the options of PLIF and TLIF by presenting their advantages and disadvantages. However, it must be emphasized that to date, there are still no well designed, prospective, double blinded, randomized-controlled trials providing evidence to distinguish the superiority of one technique over the others.

Throughout the many years since the description of PLIF by Briggs and Milligan in 1944,^5^ the practice of PLIF has evolved tremendously. With the development of more options of autologous bone grafting, newer methods of spinal segment fusion techniques, newer implants including the vast variety of cages we use today and the use of pedicle screws for posterior instrumentation, the results of spinal fusion in PLIF has improved.^6^–^9^ However, in 1982, Harms and Rolinger reported a newer technique of achieving insertion of the interbody cage packed with bone graft via the transforaminal route, termed transforaminal lumbar interbody fusion (TLIF).^10^ This created another option for surgeons in their armamentarium for treatment of patients with symptomatic spondylolisthesis and degenerative lumbar scoliosis.

## PLIF VS. TLIF

The concept of a PLIF involves positioning the patient prone on a surgical table to assume a normal lordotic position of the lumbar spine. A midline incision and retraction of paraspinal musculature are performed. In the event when posterolateral fusion across transverse processes is intended, dissection is carried further until the transverse processes are accessible. A laminectomy is performed to allow access to the central spinal canal. The visible dura is then retracted medially together with the traversing nerve roots to achieve access to the intervertebral discs. The disc is then removed piecemeal and the adjacent endplates of the vertebral bodies prepared. Insertion of cage(s) packed with morcelised bone graft is done under distraction of adjacent vertebral segments to allow compression which helps with the fusion process. This is followed by pedicle screws and rod insertion to complete the posterior construct for added stability.

The method of TLIF is similar with some exceptions. Posterolateral fusion is often not required and extensive soft tissue dissection is thus minimized. The posterior construct consisting of pedicle screws are inserted immediately after retraction of the erector spinae musculature. With a transforminal approach, only unilateral laminectomy and inferior facetectomy are required at the intended level of fusion to gain access to the intervertebral disc preserving more bony surfaces. Discectomy, end-plate preparation and insertion of cage(s) packed with morcelised bone graft are similar to PLIF. Rod insertion and final tightening under compression to achieve final posterior stabilization completes the procedure.

When comparing PLIF and TLIF, it becomes obvious that there are many similarities. Posterior approaches allow decompression of posterior spinal elements (e.g. laminotomy, laminectomy, partial facetectomy, excision of hypertrophied ligamentum flavum and discectomy) to be performed if required on other segments.^11^ Posterior instrumentation can be inser ted to allow immediate postoperative stability when bony fusion is still not established.^12^,^13^ Both PLIF and TLIF give a wider area of intervertebral bone-to-graft contact for fusion. With the interbody space bearing richer vascular supply than the posterolateral spaces, better chance of fusion is expected.^3^

The process of TLIF completely alleviates the need for posterolateral fusion in most patients. This reduces the need for lateral dissection and retraction of paraspinal musculature to expose the transverse processes and potentially reduces bleeding and post-operative pain. However, in cases of pseudoarthrosis after multiple fusion attempts, intertransverse fusion should still be a considered option. The transforaminal approach also allows access to the spinal canal more laterally, avoiding the need to expose the midline dura. This inadvertently decreases the risk of an accidental durotomy and its complications.^10^ As such, TLIF has gained wide acceptance in our current practice.

The disadvantages of TLIF such as incomplete removal of intervertebral disc and vertebral end-plate, potential occult injury to exiting nerve root, however when under experienced hands are uncommon. TLIF, being a unilateral transforaminal approach, is also unable to decompress the opposite nerve root. This may require contralateral laminotomy, which is a fairly simple procedure. The advantages of TLIF and PLIF are summarized in Table 1.

**Table 1:** Advantages and disadvantages of TLIF over PLIF

Advantages
Posterior approaches allow easy access to lamina, ligamentum flavum, facet joints
Allows posterior instrumentation
Less soft tissue/ paraspinal muscle dissection
Less removal of bony surfaces
Less exposure of midline neural structures (e.g. dura)

Disadvantages
Unilateral approach and inability to decompress opposite nerve root
Incomplete intervertebral disc removal

Our experience in ALIF/PLIF versus TLIF^14^ in a retrospective study of 53 ALIF/PLIF patients and 111 TLIF patients with lumbar degenerative disc disease demonstrates that TLIF has a shorter operative time, lesser blood loss, shorter hospital stay, and lower complications rates (51% in ALIF/PLIF fusion; 28% in TLIF). Pseudoarthrosis, wound infection and radiculopathy were the most frequent complications in this study

In another study^15^ on 52 patients with a minimum follow- up of three years, the pain relief in the visual analog scale (VAS) and the reduction of the Oswestry disability index (ODI) were significant at follow-up. There was good fusion rate of 89%. The study concluded that the TLIF technique was comparable to other interbody fusions, such as the PLIF and ALIF techniques. Also, the potential advantages of the TLIF technique included avoidance of the anterior approach, and reduction of neural complications when compared to PLIF.

## THE NEW PROCEDURE: MI-TLIF

Ever since the introduction of MI-TLIF as a new form of approach for patients with degenerative deformity of the spine, it has received widespread recognition.^16^,^17^ Its promising feature lies in the fact that TLIF can be performed with presumably fewer complications, faster recovery and better cosmesis.

Many surgeons describe variable forms of MI-TLIF techniques. Most involve localization of the relevant facet joint via fluoroscopic guidance; a paramidline incision and approach with retraction of erector spinae muscles through insertion of serial dilators until a self-retaining retractor is assembled. Facetectomy and annulotomy are performed as per open TLIF. This is followed by discectomy, preparation of adjacent vertebral endplates, insertion cage packed with morcelised bone graft. Fluoroscopic guidance is employed to insert guide wires and subsequently pedicle screws via the same incision ipsilaterally and stab incisions contralaterally. Finally, posterior stabilization is achieved with rods glided into the screw heads and tightened using set screws under compression.

The key differences in MI-TLIF compared with the open method include the exchange of longer skin incision with multiple small ones, less dissection of adjacent musculature and the employment of fluoroscopy to compensate for the technically more challenging limited access surgery. As such, it is only understandable for MI-TLIF to have certain potential advantages and disadvantages listed in Table 2. However, even with numerous studies conducted to compare open and MI-TLIF, no data has passed the scrutiny of any well designed, prospective, double blinded, randomized-controlled trials.

**Table 2:** Advantages and disadvantages of MI-TLIF over open TLIF

Advantages
Less operative blood loss
Shorter length of stay
Fewer complications
Faster recovery
Cosmetically superior

Disadvantages
Technically more difficult
Steep learning curve
More expensive

Several recent studies have shown the improvement of VAS and ODI scores in MI-TLIF. In a recent study^18^ conducted on 40 consecutive patients who underwent MI-TLIF for symptomatic spondylolisthesis, with a minimum follow- up of 24 months and a mean of 35 months, the mean preoperative ODI score was 55, decreasing to a mean of 16 postoperatively. Preoperatively, the mean leg and back pain VAS scores were 65 and 52, which improved to average of 8 and 15 respectively. In another prospective study^19^ of 34 patients with a follow-up duration ranging from six to 12 months, 17 (85%) out of 20 patients achieved decrease in mean ODI score from 57 to 18. The patients' VAS pain scores improved from 8.3 preoperatively to 1.4 after surgery.

Three recent published studies have also shown promising results of MI-TLIF over open TLIF comparing the length of surgery, length of stay, estimated blood loss and functional outcome.^20^–^22^

In the first study^20^ (n=42) who underwent TLIF for degenerative disc disease or spondylolisthesis, 21 patients underwent MI-TLIF and 21 patients underwent open TLIF. The mean follow-up was 24 months for the MI group and 34 months for the open group. The mean estimated blood loss was 194 ml for the MI-TLIF group and 505 ml for the open group. The mean length of hospital stay (LOS) was three days for the MI-TLIF group and 5.5 days for the open group. The mean modified Prolo score improved from 11 to 19 in the MI-TLIF group and from 10 to 18 in the open group. Patients in the MI-TLIF group had a shorter LOS, less estimated blood loss but there was no statistically significant difference in Prolo score improvement (*P*=0.19).

In the second study^21^ comparing 29 MI-TLIF patients and 29 open TLIF, MI-TLIF resulted in longer operative time, less blood loss, shorter hospital stay, with no difference in SF-36 scores at six months and two years. The last study^22^ of 36 patients who underwent open TLIF and MI-TLIF with an average follow-up of 22 and 24 months, there was no difference in LOS between the two groups. The MI-TLIF group resulted in a significant reduction of blood loss and had a shorter length of hospital stay. No differences were observed in postoperative pain, initial analgesia consumption, VAS or ODI between the groups. However, a steeper learning curve for the surgeons was observed for the MI-TLIF group.

Due to minimal exposure during the operation, MI-TLIF is a challenge for the inexperienced spine surgeon. Restricted by the confines of the exposed operative field, laminectomy, facetectomy, discectomy and cage insertion become technically demanding. This may translate to greater risks during the operation. It has hence been argued that the term “minimal invasive” should be termed “minimal access” instead. However, it is reasonable to believe that under good hands, with less exposure and tissue dissection, there will be reduced risk of vessel and nerve injury, and faster recovery of patients.

## CONCLUSION

It is obvious that more evidence will be required to prove whether MI-TLIF can be an alternative to, or even replace open TLIF as the standard of surgical care for degenerative lumbar spinal disease. It is certain that improved technology and infrastructure support will facilitate the process. Nonetheless, considering the pros and cons each of these surgical procedures can help in the selection of the most appropriate one, which is patient-based and individualized.
